# Prevalence and Prognostic Impact of the Coexistence of Cachexia and Sarcopenia in Patients With Chronic Liver Diseases

**DOI:** 10.1002/jcsm.70305

**Published:** 2026-05-05

**Authors:** Takatsugu Tanaka, Goki Suda, Masatsugu Ohara, Daisuke Yokoyama, Shoichi Kitano, Osamu Maehara, Tomoka Yoda, Qingjie Fu, Zijian Yang, Naohiro Yasuura, Akimitsu Meno, Takashi Sasaki, Risako Kohya, Takashi Kitagataya, Naoki Kawagishi, Masato Nakai, Takuya Sho, Shunsuke Ohnishi, Naoya Sakamoto

**Affiliations:** ^1^ Department of Gastroenterology and Hepatology, Graduate School of Medicine Hokkaido University Sapporo Japan; ^2^ Laboratory of Molecular and Cellular Medicine, Faculty of Pharmaceutical Sciences Hokkaido University Sapporo Japan

**Keywords:** cachexia, chronic liver diseases, hepatocellular carcinoma, liver cirrhosis, sarcopenia

## Abstract

**Background:**

Cachexia and sarcopenia are prevalent, inflammation‐linked syndromes in chronic liver disease that worsen outcomes. To our knowledge, their coexistence in a single chronic liver disease cohort has not been systematically examined. In this study, we evaluated the prevalence, clinical features and prognostic impact of cachexia and sarcopenia—alone and combined—in chronic liver disease.

**Methods:**

We retrospectively screened 776 patients with liver cirrhosis (LC) and/or hepatocellular carcinoma (HCC) at Hokkaido University Hospital (August 2014–May 2025). The inclusion criteria were grip strength, CT‐based muscle mass and complete clinical data, yielding 307 patients; 469 did not meet one of the inclusion criteria. Cachexia was determined following the Asian Working Group for Cachexia criteria, and sarcopenia was determined following Japan Society of Hepatology guidelines. Patients were grouped as no cachexia/sarcopenia, cachexia only, sarcopenia only or cachexia+sarcopenia. The outcomes were overall survival, time to liver‐related events and time to readmission (Kaplan–Meier and Cox‐proportional models).

**Results:**

Among 776 patients, 307 were included in the final‐analysis. Of 307 patients, 206 (67.1%) were male, the median age was 70 years (range, 19–90 years), 262 patients (85.3%) had LC and 188 patients (61.2%) had HCC. The patients were grouped as no cachexia/sarcopenia (213; 69.4%), cachexia only (54; 17.6%), sarcopenia only (17; 5.5%) and cachexia+sarcopenia (23; 7.5%). The combined group compared with the others had the lowest body mass index, psoas‐muscle‐index and grip strength (all *p* < 0.001). Overall survival (OS), liver‐related events, LC progression and readmissions were compared between 246 patients with and without cachexia or sarcopenia, after excluding those who visited the hospital on or after July 2023 and had ≤ 3 months of follow‐up. OS was shorter in the cachexia only (median 61.8 [95% CI 40.90–not reached (NR)] months, *p* = 0.046) and cachexia+sarcopenia (median 59.6 [95% CI 14.26–NR] months, *p* = 0.027) groups than in the no cachexia/sarcopenia group. Multivariable analysis showed that cachexia+sarcopenia (hazard ratio 2.48, *p* = 0.010), HCC (hazard ratio 3.40, *p* < 0.001) and diabetes mellitus (hazard ratio 1.80, *p* = 0.013) independently predicted mortality. The combined group compared with the other groups had a shorter time to liver‐related events and readmission.

**Conclusions:**

The coexistence of cachexia and sarcopenia—rather than either alone—can be used as an indicator for identifying patients with chronic liver disease at the highest risk of poor outcomes. Concurrent assessment and early, targeted interventions may improve outcomes in this population.

## 1 Introduction

The liver is central to protein, glucose, lipid, amino acid and trace element metabolism and is an essential organ for maintaining nutritional homeostasis [1]. As chronic hepatitis progresses, persistent inflammation and fibrosis lead to impaired hepatocellular function, resulting in liver cirrhosis (LC) [2]. In LC, factors such as impaired synthesis, abnormal lipid metabolism and hyperammonaemia overlap lead to sarcopenia, which is characterised by muscle mass and strength loss, via malnutrition [2, 3]. Inflammation also induces bacterial translocation and appetite loss, potentially contributing to cachexia [4]. Many patients with hepatocellular carcinoma (HCC) have LC, where paraneoplastic cytokines and tumour‐derived factors induce systemic inflammation and a hypercatabolic state [5]. This, combined with decreased appetite and metabolic alterations due to interventions, such as anticancer drug therapy, can lead to sarcopenia and cachexia [6].

Cachexia is a systemic metabolic disorder characterised by loss of body protein, muscle weakness and loss of appetite and is often associated with chronic diseases, such as cancer, chronic heart failure, chronic obstructive pulmonary disease, chronic kidney disease and chronic liver disease [7, 8]. Unlike malnutrition, cachexia is characterised by significant inflammatory responses and metabolic abnormalities, which can have a major impact on physical function and quality of life, and is associated with reduced survival rates, making the management of chronic diseases challenging [9, 10]. In liver disease, cachexia is frequently observed in patients with LC and/or HCC, both of which are associated with systemic inflammation, nutritional metabolic abnormalities and hormonal imbalances [4, 11]. Cachexia is an independent predictor of poor prognosis, contributing to worsened functional status, reduced treatment tolerance and increased mortality [12, 13, 14, 15].

In contrast, sarcopenia is a disease of old age defined as a decrease in muscle mass, muscle strength and/or physical ability associated with ageing. It often occurs simultaneously with cancer, renal dysfunction, liver disease, metabolic disorders and other conditions [16, 17]. Sarcopenia increases the risk of falls and fractures, causes movement disorders and leads to a decline in quality of life, loss of independence or the need for long‐term care, ultimately leading to death [18]. Sarcopenia is commonly observed in patients with LC and/or HCC, functioning as an independent predictor of poor prognosis and contributing to worsening functional status and increased mortality rates [3, 19].

Although the definitions of cachexia and sarcopenia overlap, cachexia is characterised by weight loss, whereas weight loss is not a characteristic of sarcopenia, making them distinct clinical concepts [7, 16, 18]. However, no studies have evaluated the simultaneous occurrence of cachexia with sarcopenia in individuals with liver disease (including LC and/or HCC) nor have they reported their prevalence, the proportion of cases with both diseases or their characteristics, including overall survival (OS) rates.

The purpose of this study was to clarify the proportion and characteristics of patients with chronic liver disease who have cachexia, sarcopenia or both (overlapping condition) and to examine their impact on prognosis, including OS.

## 2 Methods

### 2.1 Patients and Study Design

In this retrospective study, we screened patients with LC and/or HCC who visited Hokkaido University Hospital between August 2014 and May 2025. Patients were included if they had appropriate clinical information, such as grip strength measurements, for analysis and if muscle mass could be measured using a computed tomography (CT) scan. Patients were excluded if they lacked grip strength measurements or CT scan results or if they did not have appropriate clinical information. Data on patient sex, age, weight, body mass index (BMI), grip strength, presence or absence of appetite loss, history of diabetes mellitus, cause of liver disease, presence or absence of LC, liver function (based on the Child–Pugh class), presence or absence of oral branched‐chain amino acids (BCAA) administration, presence or absence of HCC, date of death or last day of survival, presence or absence of liver‐related events and their details (hepatic encephalopathy, oesophageal/gastric variceal rupture, worsening ascites, spontaneous bacterial peritonitis, and portal vein thrombosis), presence or absence of readmission, and the duration until readmission were collected. Patients with LC who had ascites, variceal bleeding, hepatic encephalopathy and jaundice were defined as having decompensated LC [20], and the presence or absence of progression from compensated to decompensated LC and duration until progression were also measured.

The primary endpoint was to assess the co‐occurrence of sarcopenia and cachexia at the individual patient level within the same cohort of patients with liver disease; to determine the proportions and characteristics of patients with (1) both conditions, (2) either condition alone or (3) neither; and to evaluate their impact on prognosis, including OS. Key secondary endpoints were time to liver‐related events, progression from compensated to decompensated LC and time to hospital readmission. Readmission was defined as the first admission after the entry date.

This study adhered to the ethical principles outlined in the Declaration of Helsinki. The Hokkaido University Hospital ethics committee approved the study protocol (approval numbers: 023‐0060). Participation in the study was contingent upon patients providing written informed consent to participate. Participants were also included under an IRB‐approved opt‐out procedure for individuals who had previously granted a broad institutional consent but had not signed a study‐specific consent form and did not decline participation.

### 2.2 Skeletal Muscle Mass Calculation Using CT Imaging

In this study, skeletal muscle mass was assessed using the psoas muscle index (PMI), a previously validated method for evaluating muscle atrophy [14, 21]. PMI was calculated from CT images as follows: The sum of the L3 level cross‐sectional area of the right and left psoas muscle mass was identified by manual tracing and divided by height squared (cm^2^/m^2^).

Muscle atrophy was defined as PMI values below 3.74 cm^2^/m^2^ in men and below 2.29 cm^2^/m^2^ in women, as previously reported [21].

### 2.3 Definition of Cachexia and Sarcopenia

Cachexia was defined based on the consensus report of the Asian Working Group for Cachexia [1]. The diagnosis required the presence of an underlying chronic disease along with at least one of the following: (A) unintentional weight loss of ≥ 2% over a period of 3–6 months or (B) BMI < 21 kg/m^2^ and at least one of the following: (I) reduced handgrip strength (men < 28 kg, women < 18 kg), (II) appetite loss or (III) elevated serum C‐reactive protein (CRP) level (> 0.5 mg/dL) (Supporting Information Figure S1).

According to the Japan Society of Hepatology (JSH) guidelines for sarcopenia in liver disease [22], low muscle strength was defined as handgrip strength < 28 kg for men and < 18 kg for women. Sarcopenia was diagnosed based on the presence of both reduced muscle strength and mass, in accordance with the JSH criteria (Supporting Information Figure S1).

### 2.4 Statistical Analysis

For categorical variables, the chi‐squared or Fisher's exact test was applied as appropriate. Continuous variables were compared using the Mann–Whitney *U* test. For tests involving three or more groups, the Bonferroni correction method was used. Analyses of OS, event‐free survival and time to readmission included only patients enrolled after July 2023, with an observation period of 3 or more months, and who were censored at the time of final survival. OS, event‐free survival and time to readmission were estimated using the Kaplan–Meier method, and differences between groups were assessed using the log‐rank test. Cox proportional hazards regression models were used to identify prognostic factors for these endpoints. Variables with *p* < 0.10 in univariate analyses were entered into multivariable models. For OS and time to liver‐related events, we constructed three prespecified multivariable Cox proportional models to address potential multicollinearity among liver function indices and to assess the robustness of liver function/nutritional markers: Model A included the Child–Pugh score (or class), Model B included serum albumin as a continuous variable (per 1 g/dL increase) and Model C included the modified albumin–bilirubin (ALBI; mALBI) grade (dichotomised as grade 1–2a vs. 2b–3). All other covariates were identical across the three models. A two‐sided *p* value < 0.05 was considered statistically significant. All statistical analyses were performed using Prism 9.41 (GraphPad Software, La Jolla, California) and EZR (Saitama Medical Center, Jichi Medical University, Saitama, Japan).

## 3 Results

### 3.1 Patient Characteristics

**FIGURE 1:**
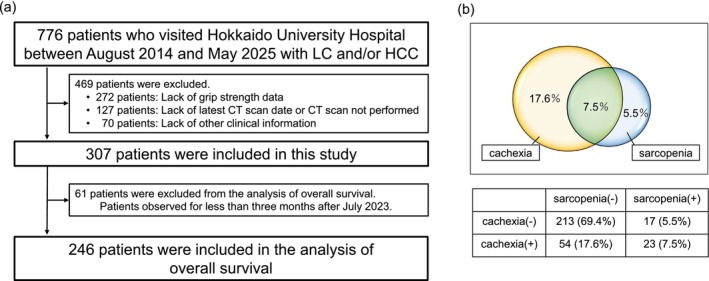
Study flow and prevalence of cachexia and sarcopenia. (a) Flowchart of the study. (b) Rates of cachexia and sarcopenia in this study. HCC, hepatocellular carcinoma; LC, liver cirrhosis.

We screened 776 patients with LC and/or HCC between August 2014 and May 2025; 272 lacked grip strength data, 127 lacked CT scan data and 70 had insufficient clinical data and were subsequently excluded. The final study included 307 patients (Figure 1a).

Baseline patient characteristics are shown in Table 1 and Figure 1b. In this study, 77 patients (25.1%) had cachexia, while 40 patients (13.0%) had sarcopenia. Of all patients, 206 (67.1%) were male and the median age was 70 years (range, 19–90 years). The aetiological agents of 130, 66, 72 and 39 patients were virus, alcohol, metabolic dysfunction‐associated steatohepatitis and other causes, respectively. A total of 262 patients (85.3%) had LC, and 211 (68.7%), 85 (27.7%) and 11 (3.6%) patients were classified as Child–Pugh class A, B and C, respectively. A total of 188 patients (61.2%) had HCC, and according to the Barcelona Clinic Liver Cancer (BCLC) stages, 70 (37.2%), 53 (28.2%), 33 (17.6%) and 32 (17.0%) were classified as BCLC stages 0, A, B and C, respectively. The median serum albumin level was 3.8 g/dL (range, 2.2–5.0 g/dL) and CRP level was 0.11 mg/dL (range, 0.02–6.48 mg/dL).

**TABLE 1:** Baseline patient characteristics and comparison between patients with and without cachexia/sarcopenia.

Sample characteristics	Overall (*n* = 307)	No cachexia/sarcopenia (*n* = 213)	Cachexia only (*n* = 54)	Sarcopenia only (*n* = 17)	Cachexia+sarcopenia (*n* = 23)	*p*
Age, years	70 (19–90)	69 (19–90)	73 (39–85)	70 (58–86)	73 (36–84)	0.269
Sex, male/female	206/101	146/67	36/18	14/3	10/13	0.050
Diabetes mellitus, no/yes	195/112	146/67	28/26	7/10	14/9	0.026
Intake of BCAA supplements, no/yes	208/99	147/66	37/17	10/7	14/9	0.732
Aetiology						0.317
Virus	130 (42.3%)	99	20	5	6	
HBV	77 (25.1%)	64	9	2	2	
HCV	53 (17.3%)	35	11	3	4	
Alcohol	66 (21.5%)	47	12	2	5	
MASH	72 (23.5%)	45	14	6	7	
Others	39 (12.7%)	22	8	4	5	
HBV antiviral therapy (among HBV‐infected patients)						0.629
On nucleos(t)ide analogue	67	54	9	2	2	
Without nucleos(t)ide analogue therapy	10	10	0	0	0	
History of HCV treatment						0.499
SVR after DAA/IFN therapy	35	25	7	1	2	
Without SVR	18	10	4	2	2	
Liver cirrhosis, no/yes	45/262	25/188	12/42	4/13	4/19	0.163
Child–Pugh class						0.146
A	211 (68.7%)	154	32	12	13	
B	85 (27.7%)	54	19	3	9	
C	11 (3.6%)	5	3	2	1	
HCC, no/yes	119/188	89/124	12/42	9/8	9/14	0.037
BCLC stage						0.121
0	70 (37.2%)	51	12	4	3	
A	53 (28.2%)	37	12	1	3	
B	33 (17.6%)	22	5	2	4	
C	32 (17.0%)	14	13	1	4	
History of systemic therapy for HCC before enrollment, no/yes	159/29	106/18	35/7	6/2	12/2	0.872
MKI	18	12	4	2	0	
ICI‐based therapy	3	1	1	0	1	
MKI + ICI‐based therapy	8	5	2	0	1	
Biochemical analysis						
Platelet, ×10^4^/μL	12.10 (1.90–66.50)	11.70 (2.80–46.40)	13.30 (1.90–40.00)	12.00 (5.20–39.70)	13.60 (6.30–66.50)	0.146
T‐Bil (mg/dL)	0.90 (0.30–18.40)	0.90 (0.40–6.70)	0.85 (0.40–18.40)	1.00 (0.30–2.70)	0.80 (0.30–2.90)	0.451
AST, IU/L	33.00 (10.00–601.00)	32.0 (10.0–601.0)	35.0 (20.0–155.0)	39.0 (20.0–100.0)	38.0 (14.0–108.0)	0.032
ALT, IU/L	23.00 (5.00–613.00)	23.0 (5.0–613.0)	25.0 (7.0–214.0)	23.0 (11.0–61.0)	22.0 (7.0–72.0)	0.709
Serum albumin, g/dL	3.80 (2.20–5.00)	3.90 (2.20–5.00)	3.80 (2.20–4.70)	3.60 (2.50–4.40)	3.70 (2.20–4.90)	0.053
Fib‐4 index	3.93 (0.38–31.20)	3.88 (0.51–18.67)	4.12 (1.24–31.20)	4.11 (1.24–13.74)	3.83 (0.38–11.37)	0.700
mALBI grade						0.283
1	135 (44.0%)	98	22	6	9	
2a	66 (21.5%)	50	10	3	3	
2b	90 (29.3%)	58	16	6	10	
3	16 (5.2%)	7	6	2	1	

*Note:* Data are presented as numbers or medians (range).

Abbreviations: ALT, alanine aminotransferase; AST, aspartate aminotransferase; BCAA, branched‐chain amino acids; BCLC, Barcelona Clinic Liver Cancer; DAA, direct‐acting antiviral; HBV, hepatitis B virus; HCV, hepatitis C virus; HCC, hepatocellular carcinoma; ICI, immune checkpoint inhibitor; IFN, interferon; mALBI, modified albumin–bilirubin; MASH, metabolic dysfunction‐associated steatohepatitis; MKI, multikinase inhibitor; T‐Bil, total bilirubin; SVR, sustained virologic response.

### 3.2 Prevalence of Sarcopenia and Cachexia and Characteristics of Patients With and Without Cachexia/Sarcopenia

We divided the entire cohort into four groups based on the presence or absence of cachexia and sarcopenia in chronic liver disease and compared them. Of the 307 patients, 213 (69.4%) had no cachexia/sarcopenia, 54 (17.6%) had only cachexia, 17 (5.5%) had only sarcopenia and 23 (7.5%) had cachexia+sarcopenia. The characteristics are shown in Table 1.

The median age and sex were not significantly different among the four groups (*p* = 0.269 and 0.050, respectively). The numbers of patients with diabetes mellitus were 67 (31.5%), 26 (48.1%), 10 (58.8%) and 9 (39.1%) in the no cachexia/sarcopenia, cachexia only, sarcopenia only and cachexia+sarcopenia groups, respectively, with a significant difference among the four groups (*p* = 0.026). Bonferroni correction showed significant differences between the no cachexia/sarcopenia and the cachexia only or sarcopenia only groups (*p* = 0.032 and 0.042, respectively). Furthermore, no significant differences were observed among the groups regarding aetiology, presence of LC or Child–Pugh class (*p* = 0.317, 0.163 and 0.146, respectively). The numbers of patients with HCC were 124 (58.2%), 42 (77.8%), 8 (47.1%) and 14 (60.9%) for the no cachexia/sarcopenia, cachexia only, sarcopenia only and cachexia+sarcopenia groups, respectively, with a significant difference observed among the four groups (*p* = 0.037). Bonferroni correction showed significant differences between the no cachexia/sarcopenia and cachexia only groups (*p* = 0.013) and between the cachexia only and sarcopenia only groups (*p* = 0.034). However, no significant difference was observed in the BCLC stage. Among patients with HCC (Supporting Information Table S1), the prevalence of cachexia, sarcopenia and their coexistence did not differ significantly by the stage of HCC (early vs. advanced/progressive) or use of multikinase inhibitors or MTA/immune checkpoint inhibitor use (Supporting Information Figure S2). Aspartate aminotransferase levels were 32.0 IU/L, 35.0 IU/L, 39.0 IU/L and 38.0 IU/L in the no cachexia/sarcopenia, cachexia only, sarcopenia only and cachexia+sarcopenia groups, respectively, with a significant difference observed among the four groups (*p* = 0.032). However, Bonferroni correction revealed no significant differences between any two groups within the four groups.

The characteristics of cachexia‐ and sarcopenia‐related items are shown in Table 2. The median BMI was 25.36, 23.59, 24.28 and 19.12 kg/m^2^ for the no cachexia/sarcopenia, cachexia only, sarcopenia only and cachexia+sarcopenia groups, respectively, with a significant difference among the four groups (*p* < 0.001). Bonferroni correction showed significant differences between the no cachexia/sarcopenia and cachexia only groups (*p* = 0.018) and between the cachexia+sarcopenia group and the no cachexia/sarcopenia or cachexia only groups (*p* < 0.001 and 0.019), respectively.

**TABLE 2:** Baseline patient characteristics and comparison between patients with and without cachexia/sarcopenia (cachexia/sarcopenia related items).

Sample characteristics	Overall (*n* = 307)	No cachexia/sarcopenia (*n* = 213)	Cachexia only (*n* = 54)	Sarcopenia only (*n* = 17)	Cachexia+sarcopenia (*n* = 23)	*p*
BMI, kg/m^2^	24.69 (14.86–42.56)	25.36 (15.16–36.71)	23.59 (14.97–42.56)	24.28 (21.63–29.25)	19.12 (14.86–36.14)	< 0.001
Decreased grip strength, no/yes	177/130	156/57	21/33	0/17	0/23	< 0.001
Appetite loss, no/yes	266/41	198/15	37/17	16/1	15/8	< 0.001
PMI, cm^2^/m^2^	3.99 (0.71–9.23)	4.30 (1.41–9.23)	4.04 (0.71–7.20)	2.93 (0.91–3.73)	2.13 (1.05–3.71)	< 0.001
Muscle atrophy, no/yes	222/85	176/37	46/8	0/17	0/23	< 0.001
Biochemical analysis						
CRP, mg/dL	0.11 (0.02–6.48)	0.08 (0.02–3.17)	0.66 (0.02–6.48)	0.13 (0.02–4.54)	0.25 (0.02–4.62)	< 0.001

*Note:* Data are presented as numbers or medians (range).

Abbreviations: BMI, body mass index; CRP, C‐reactive protein; PMI, psoas muscle mass index.

The number of patients with decreased grip strength was 57 (26.7%), 33 (61.1%), 17 (100%) and 23 (100%) in the no cachexia/sarcopenia, cachexia only, sarcopenia only and cachexia+sarcopenia groups, respectively, with a significant difference observed among the four groups (*p* < 0.001). Bonferroni correction revealed significant differences in all two‐group comparisons (*p* = 0.001 for the comparison between cachexia only and sarcopenia only groups and *p* < 0.001 for all other comparisons). The number of patients with loss of appetite was 15 (7.0%), 17 (31.5%), 1 (5.9%) and 8 (34.8%) in the no cachexia/sarcopenia, cachexia only, sarcopenia only and cachexia+sarcopenia, respectively, with a significant difference observed among the four groups (*p* < 0.001). Bonferroni correction showed significant differences between the no cachexia/sarcopenia group and the cachexia only or cachexia+sarcopenia group (*p* < 0.001 in both). Median PMI was 4.30, 4.04, 2.93 and 2.13 for the no cachexia/sarcopenia, cachexia only, sarcopenia only and cachexia+sarcopenia groups, respectively, with a significant difference among the four groups (*p* < 0.001). Bonferroni correction showed significant differences between the no cachexia/sarcopenia group and the sarcopenia only or cachexia+sarcopenia groups (*p* < 0.001 in both) and between the cachexia only group and the sarcopenia only or cachexia+sarcopenia group (*p* = 0.001, < 0.001). The number of patients with muscle atrophy was 37 (17.4%), 8 (14.8%), 17 (100%) and 23 (100%) for the no cachexia/sarcopenia, cachexia‐only, sarcopenia‐only and cachexia+sarcopenia groups, respectively, with a significant difference observed among the four groups (*p* < 0.001). Bonferroni correction showed significant differences in both the two groups with sarcopenia and the two groups without sarcopenia. CRP was 0.08 mg/dL, 0.66 mg/dL, 0.13 mg/dL and 0.25 mg/dL for the no cachexia/sarcopenia, cachexia only, sarcopenia only and cachexia+sarcopenia groups, respectively, with a significant difference observed among the four groups (*p* < 0.001). Bonferroni correction showed significant differences between the no cachexia/sarcopenia and cachexia only groups (*p* < 0.001).

### 3.3 Comparative Analysis of OS Between Patients With and Without Cachexia or Sarcopenia

We conducted a comparative analysis of OS, clinical events, LC progression and readmission between patients with and without cachexia or sarcopenia. For this analysis, patients who visited the hospital from July 2023 onwards with ≤ 3 months of follow‐up were excluded because the observation period was insufficient for reliable outcome assessment (Figure 1a).

**FIGURE 2:**
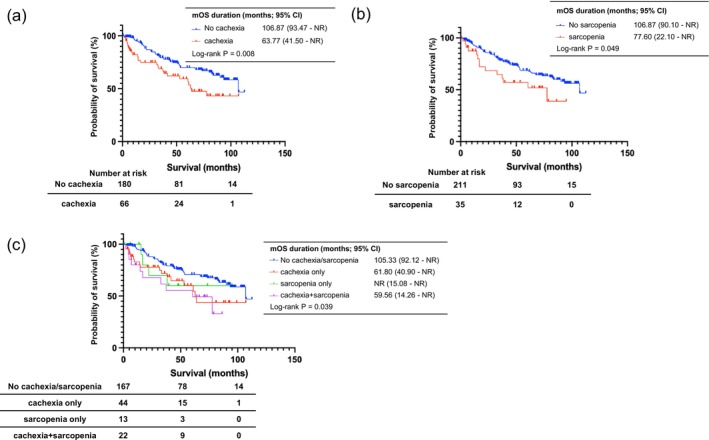
Overall survival according to cachexia and sarcopenia status. (a) Comparison of the overall survival in patients with cachexia. (b) Comparison of the overall survival in patients with sarcopenia. (c) Kaplan–Meier estimates of overall survival, stratified by the presence or absence of cachexia and sarcopenia. CI, confidence interval; mOS, median overall survival; NR, not reached.

We evaluated the impact of cachexia or sarcopenia on the prognosis of patients with chronic liver disease in the entire cohort. As shown in Figure 2a,b, patients with only cachexia and only sarcopenia had significantly shorter median OS than those had by patients without either of them (cachexia: 63.77 months [95% confidence interval (CI) 41.50–not reached (NR) months] vs. 106.87 months [95% CI 93.47–NR months], *p* = 0.008; sarcopenia: 77.60 months [95% CI 22.10–NR months] vs. 106.87 months [95% CI 90.10–NR months], *p* = 0.049).

Figure 2c, which compares OS among the four groups, showed significant differences (*p* = 0.039). The OS in the cachexia only and cachexia+sarcopenia groups was significantly shorter than that in no cachexia/sarcopenia group (61.80 months [95% CI 40.90–NR months] and 59.56 months [95% CI 14.26–NR months] vs. 105.33 months [95% CI 92.12–NR months], *p* = 0.046 and 0.027, respectively).

Subsequent univariate and multivariate Cox regression analyses revealed that the presence of sarcopenia and cachexia (hazard ratio [HR] 2.479 [95% CI 1.241–4.948], *p* = 0.010), history of diabetes mellitus (HR 1.796 [95% CI 1.134–2.846], *p* = 0.013) and HCC (HR 3.401 [95% CI 1.922–6.020], *p* < 0.001) were identified as independent risk factors for OS (Model A) (Table 3). Similar results were observed in Models B and C.

**TABLE 3:** Factors associated with overall survival in patients with liver disease: Cox proportional hazards analysis.

	Univariable analysis		Multivariable analysis					
	HR (95% CI)	*p*	HR (95% CI) Model A	*p*	HR (95% CI) Model B	*p*	HR (95% CI) Model C	*p*
Age, years	1.025 (1.001–1.049)	0.043	1.012 (0.987–1.038)	0.346	1.015 (0.990–1.041)	0.254	1.019 (0.993–1.045)	0.153
Sex, women	0.928 (0.576–1.494)	0.757						
No cachexia/sarcopenia	1 (Reference)		1 (Reference)		1 (Reference)		1 (Reference)	
Cachexia only	1.738 (0.996–3.034)	0.052	1.420 (0.809–2.495)	0.222	1.213 (0.689–2.136)	0.503	1.318 (0.750–2.317)	0.337
Sarcopenia only	1.499 (0.539–4.173)	0.438						
Cachexia+sarcopenia	2.335 (1.174–4.644)	0.016	2.479 (1.241–4.948)	0.010	2.626 (1.297–5.316)	0.007	2.672 (1.325–5.387)	0.006
History of diabetes mellitus	1.642 (1.047–2.576)	0.031	1.796 (1.134–2.846)	0.013	1.759 (1.109–2.790)	0.016	1.707 (1.076–2.708)	0.023
Aetiology, virus	1 (Reference)							
Alcohol	1.073 (0.606–1.902)	0.808						
MASH	1.352 (0.761–2.401)	0.304						
Others	0.889 (0.375–2.107)	0.790						
Child–Pugh class A versus B, C	1.384 (0.876–2.186)	0.163						
HCC	3.359 (1.939–5.821)	< 0.001	3.401 (1.922–6.020)	< 0.001	5.152 (2.769–9.586)	< 0.001	4.219 (2.352–7.567)	< 0.001
Serum albumin, g/dL	0.520 (0.369–0.733)	< 0.001			0.365 (0.242–0.551)	< 0.001		
mALBI grade 1–2a versus 2b–3	2.055 (1.314–3.214)	0.002					2.744 (1.718–4.384)	< 0.001

Abbreviations: CI, confidence interval; HR, hazard ratio; HCC, hepatocellular carcinoma; mALBI, modified albumin–bilirubin; MASH, metabolic dysfunction‐associated steatohepatitis.

In a sensitivity analysis restricted to non‐HCC patients, the coexistence of sarcopenia and cachexia remained significantly associated with poorer OS (Supporting Information Table S6; Supporting Information Figure S3).

### 3.4 Comparative Analysis of Liver‐Related Events, Progression of LC and Readmissions Between Patients With and Without Cachexia/Sarcopenia

**FIGURE 3:**
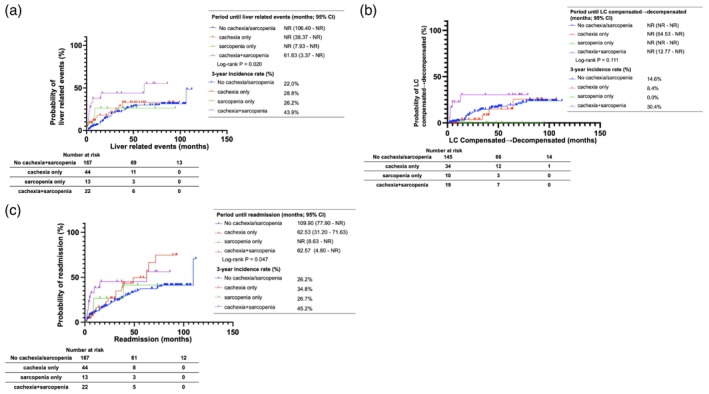
Liver‐related outcomes according to cachexia and sarcopenia status. (a) Kaplan–Meier estimates of period until liver‐related events, stratified by the presence or absence of cachexia and sarcopenia. (b) Kaplan–Meier estimates of period until LC compensated → decompensated, stratified by the presence or absence of cachexia and sarcopenia. (c) Kaplan–Meier estimates of period until readmission, stratified by the presence or absence of cachexia and sarcopenia. CI, confidence interval; NR, not reached.

We examined the presence or absence of liver‐related events and their characteristics, the breakdown of LC (compensated, decompensated, or transition from compensated to decompensated), and the presence or absence of readmission for the four groups (Supporting Information Table S4). The presence or absence of liver‐related events, breakdown of LC, and presence or absence of readmission were not significantly different among the four groups. The periods until the onset of liver‐related events, transition from compensated to decompensated LC and period until readmission due to liver‐related causes were compared and analysed and are presented in Figure 3a, b, and c, respectively. Significant differences were observed among the four groups in the duration until the onset of liver‐related events and until readmission due to liver‐related events (*p* = 0.020 and 0.047, respectively). However, regarding the progression from compensated to decompensated LC, no cases of progression were recorded in the sarcopenia group, and no statistically significant difference was observed among the remaining three groups (*p* = 0.111).

Subsequently, univariate and multivariate Cox regression analyses were performed to assess factors associated with the time to liver‐related events and time to readmission. Sarcopenia+cachexia (HR 2.149 [95% CI 1.007–4.586], *p* = 0.048), history of diabetes (HR 2.226 [95% CI 1.302–3.806], *p* = 0.003), and Child–Pugh class B and C relative to class A (HR 2.628 [95% CI 1.550–4.455], *p* < 0.001) were identified as independent risk factors for time to liver‐related events (Model A; Table 4). Similar results were observed in Models B and C.

**TABLE 4:** Comparison of the duration of liver‐related events between patients with and without cachexia and sarcopenia using Cox proportional hazards analysis.

	Univariable analysis		Multivariable analysis					
	HR (95% CI)	*p*	HR (95% CI) Model A	*p*	HR (95% CI) Model B	*p*	HR (95% CI) Model C	*p*
Age, years	0.974 (0.956–0.992)	0.005	0.982 (0.960–1.005)	0.116	0.976 (0.954–1.000)	0.049	0.979 (0.957–1.001)	0.066
Sex, women	1.538 (0.936–2.527)	0.090	1.297 (0.736–2.288)	0.369	1.306 (0.734–2.325)	0.363	1.307 (0.741–2.304)	0.355
No cachexia/sarcopenia	1 (Reference)		1 (Reference)		1 (Reference)		1 (Reference)	
Cachexia only	1.282 (0.656–2.507)	0.467						
Sarcopenia only	1.240 (0.383–4.017)	0.720						
Cachexia+sarcopenia	2.918 (1.453–5.860)	0.003	2.149 (1.007–4.586)	0.048	2.812 (1.319–5.994)	0.007	2.848 (1.333–6.083)	0.007
History of diabetes mellitus	2.458 (1.502–4.025)	< 0.001	2.226 (1.302–3.806)	0.003	2.160 (1.261–3.699)	0.005	2.234 (1.308–3.817)	0.003
Aetiology, virus	1 (Reference)		1 (Reference)		1 (Reference)		1 (Reference)	
Alcohol	1.437 (0.743–2.779)	0.281						
MASH	2.351 (1.279–4.320)	0.006	1.194 (0.613–2.324)	0.603	1.116 (0.596–2.239)	0.669	1.512 (0.786–2.906)	0.215
Others	1.723 (0.740–4.012)	0.207						
Child–Pugh class A versus B, C	3.275 (1.991–5.387)	< 0.001	2.628 (1.550–4.455)	< 0.001				
HCC	0.661 (0.402–1.086)	0.102						
Serum albumin, g/dL	0.269 (0.181–0.398)	< 0.001			0.280 (0.219–0.420)	< 0.001		
mALBI grade 1–2a versus 2b–3	3.972 (2.398–6.579)	< 0.001					4.186 (2.460–7.122)	< 0.001

Abbreviations: CI, confidence interval; HR, hazard ratio; HCC, hepatocellular carcinoma; mALBI, modified albumin–bilirubin; MASH, metabolic dysfunction‐associated steatohepatitis.

In the non‐HCC cohort, the coexistence of sarcopenia and cachexia was also significantly associated with a shorter time to liver‐related events (Supporting Information Table S7; Supporting Information Figure S3).

In contrast, with respect to readmission, a history of diabetes (HR 1.774 [95% CI 1.096–2.872], *p* = 0.020) and Child–Pugh class B and C relative to class A (HR 1.863 [95% CI 1.160–2.993], *p* = 0.010) were identified as independent risk factors. The presence or absence of cachexia and sarcopenia was not an independent risk factor (Model A; Supporting Information Table S5).

## 4 Discussion

This study is the first to examine the prevalence, survival rate, event incidence and readmission rates of cachexia and sarcopenia in patients with chronic liver disease. The main findings are as follows: (1) The no cachexia/sarcopenia, cachexia only, sarcopenia only and cachexia+sarcopenia groups accounted for 69.4%, 17.6%, 5.5% and 7.5% of the cohort, respectively. (2) Compared with the group without cachexia or sarcopenia, only the cachexia+sarcopenia group was associated with mortality. (3) Compared with the group without cachexia or sarcopenia, no group showed an association with progression from compensated to decompensated LC or readmission, but only the group with concurrent cachexia and sarcopenia showed an association with liver‐related event incidence. Instead of separate assessments of sarcopenia and cachexia, clinicians should undertake concurrent evaluations of both in patients with chronic liver disease, as intensive interventions may be warranted.

The prevalence of cachexia in the study population was 25.1%, and the prevalence of sarcopenia was 13.0%. Previous reports have indicated that the prevalence of cachexia in patients with HCC was 23.7% [11] and in those with LC was 28.0% [23] while the prevalence of sarcopenia was 42.0% in patients with HCC [24] and 33% in patients with LC [25]. Compared with the prevalence rates in this study, the prevalence rate of cachexia is generally consistent with previous reports, but the prevalence rate of sarcopenia is lower than the previously reported rate. When prevalence rates were classified into four groups based on the presence or absence of cachexia and sarcopenia, the cachexia only, sarcopenia only and cachexia+sarcopenia groups accounted for 17.6%, 5.5% and 7.5%, respectively. We found that approximately 30% of patients with cachexia had concomitant sarcopenia, while approximately 60% of patients with sarcopenia had concomitant cachexia. There are no previous reports on liver disease, but in previous reports targeting older patients with chronic heart failure, the prevalence rates were 21.7%, 11.6% and 11.0% for the cachexia only, sarcopenia only and cachexia+sarcopenia groups, respectively [26], which is consistent with the finding that the cachexia only group had the highest prevalence rate. Cachexia is characterised by the persistent loss of adipose tissue and skeletal muscle mass due to inflammatory secretory factors and tumours caused by LC or HCC [11]. However, muscle weakness is not necessarily a diagnostic requirement [7]. Sarcopenia is caused by muscle atrophy associated with disease progression [11, 27], and loss of muscle mass is a diagnostic criterion [22]. The prevalence of cachexia in patients with sarcopenia is high, suggesting that cachexia may occur earlier than sarcopenia.

Kotoh et al. reported that an ALBI score of −2.18 predicts a nonprotein respiratory quotient (npRQ) of < 0.85, a threshold indicative of impaired substrate oxidation and malnutrition, and this value approximates the mALBI grade 2b cutoff [28]. Although npRQ was not available in our cohort, our additional analyses demonstrated that both serum albumin (modelled continuously) and the composite ALBI/mALBI framework were independently associated with OS. The consistent prognostic separation observed for mALBI grade 2b–3 suggests that ALBI/mALBI in conjunction with hepatic functional reserve may capture nutrition‐related vulnerability in liver disease, potentially providing clinically meaningful risk stratification beyond albumin alone. Future prospective studies incorporating standardised nutritional assessments are warranted. In patients with LC and/or HCC, low serum albumin may have clinical implications beyond liver function and nutritional status. A previous study linked hypoalbuminaemia to portal venous thrombosis and suggested a mechanistic role of albumin in regulating platelet activation and haemostasis [29], which may partly contribute to adverse liver‐related outcomes.

In this study, the comparison of OS between cachexia and sarcopenia showed that the prognosis was poor in both groups, which is similar to previous reports [3, 11, 19, 23]. In contrast, the comparison of OS among the four groups categorised by the presence or absence of cachexia and sarcopenia showed that the prognosis was significantly poorer in the cachexia only and cachexia+sarcopenia groups than in the no cachexia/sarcopenia group. In the Cox proportional hazards regression analysis, the cachexia+sarcopenia group was identified as a prognostic factor. These results are consistent with previously reported findings in patients with chronic heart failure, which demonstrated that a decrease in skeletal muscle mass and fat contributes to increased mortality [26]. A comparison of background factors in this study (Supporting Information Table S2) showed that the cachexia+sarcopenia group had significantly lower BMI and PMI, suggesting a decrease in skeletal muscle mass and fat mass. This result is consistent with previous studies on liver disease, such as reports indicating that female patients with LC who have low subcutaneous fat have a poor prognosis [30]. Previous reports have also shown that patients with HCC who have low BMI have poor prognosis [31] and have indicated that the crude mortality rate in obese patients with LC is lower than that in non‐obese patients with LC [32]. The reason for the association of reduced muscle and fat mass with mortality is unclear; however, it may be related to a phenomenon known as the ‘obesity paradox’. Adipocyte‐derived substances, such as leptin and interleukin‐10, have immunomodulatory effects, suppress inflammatory responses and potentially improve host survival in severe illness [32]. Although leptin increases with worsening liver function, its expression is directly proportional to body fat percentage [33]. Reduced leptin expression in patients with reduced body fat percentage may contribute to increased inflammation. In addition, in LC, lipids are involved in inflammation as intercellular signals, and a decrease in adipose tissue may exacerbate inflammation and organ damage through abnormalities in circulating lipids, contributing to inflammation in LC [34], which may also be related to mortality.

No significant differences were observed among the four groups in event incidence, LC breakdown (compensated, decompensated and transition from compensated to decompensated) or readmission rate. However, the cachexia+sarcopenia group had a significantly worse prognosis than that observed in the no cachexia/sarcopenia group in terms of time to event onset and to readmission. Cox proportional hazards regression analysis revealed no significant differences in readmission rate among the four groups, but the cachexia+sarcopenia group showed a tendency to be associated with earlier event onset. Previous studies have shown that sarcopenia increases the risk of hepatic encephalopathy due to decreased ammonia metabolism caused by reduced skeletal muscle mass [35], increases the prevalence of LC‐related complications [36] and is an independent predictor of readmission [37]. Although no studies have demonstrated a direct increase in events or readmissions in cachexia, research has suggested that decreased nutrition and muscle mass, reflecting decreased liver function associated with cachexia, increase the frequency of hospitalisation and risk of readmission [11]. Although there were no significant differences in event incidence or readmission rate, the prevalence tended to be higher in the cachexia+sarcopenia group than in the other groups, which is consistent with previously reported trends, as cachexia and sarcopenia tend to be more common. Regarding the time to event onset, time to transition from compensated to decompensated LC, and time to readmission, previous reports have shown that cachexia associated with LC is associated with a significantly higher 30‐day readmission rate [38]. Sarcopenia associated with LC is a predictor of ascites and the associated decompensation of LC and is associated with an increased 30‐day readmission rate [39]. Decreased grip strength, a diagnostic indicator of sarcopenia, has been reported as an independent factor in the development of overt hepatic encephalopathy [40]. In this study, significant differences were observed in the time to event onset and time to readmission, and having cachexia+sarcopenia was identified as a factor associated with the time to event onset. However, there was no significant difference in the time to transition from compensated to decompensated LC among the four groups, and the multivariate analysis of the time to readmission revealed no associated factors among the group conditions. Owing to the limited number of cachexia and sarcopenia cases, the results for factors that showed significant differences are considered consistent with previous reports. However, the number of cases in the sarcopenia only group was 13, and the number of cases that progressed was also small. The results in our study may differ from previous reports. Although no factors were related to the readmission rate among the four groups, a statistically significant difference was observed in the Kaplan–Meier curve, and the time until readmission was shorter in the cachexia+sarcopenia group than in the other groups, demonstrating a trend similar to previous reports.

This study provides new evidence suggesting that the coexistence of cachexia and sarcopenia in patients with chronic liver disease is a strong predictor of mortality and event incidence. However, this study has several limitations, including its single‐centre retrospective design and relatively small sample size. In particular, the relatively short observation period may not have adequately captured the occurrence of events, readmission and the transition from compensated to decompensated LC. We could not adjust for supportive interventions (such as nutritional intervention and exercise therapy) because these data were not consistently recorded. Large‐scale, prospective and longer‐term observational studies are needed to verify these findings.

## 5 Conclusion

This study is the first to investigate the characteristics, prevalence, survival, event incidence and readmission rates of cachexia co‐occurring with sarcopenia in patients with chronic liver disease. While neither condition alone was associated with mortality or clinical deterioration, the coexistence of these conditions significantly predicted decreased survival and increased event incidence. These findings highlight the importance of assessing both cachexia and sarcopenia, rather than assessing them separately, in patients with chronic liver disease. Early identification of patients with both syndromes may improve risk stratification and guide future intervention strategies.

## Funding

This study was supported in part by grants from the Japan Agency for Medical Research and Development (AMED; Grant Nos. JP25fk0210126, JP25fk0310545, JP24fk0210121, JP25fk0210142, JP25fk0310551, JP25fk0210123, JP25fk0310535, JP25fk0210157, JP25fk0310543, JP25fk0210172, JP25fk0210174 and JP25fk0210143). The sponsor had no role in study design; in the collection, analysis and interpretation of data; in the writing of the report; or in the decision to submit the article for publication.

## Ethics Statement

The ethics committee of Hokkaido University Hospital verified that the study protocol (IRB numbers: 023‐0060) adhered to the ethical guidelines of the Declaration of Helsinki.

## Consent

Inclusion in the study was granted to patients who provided written informed consent or did not expressly refuse to participate. The ethics committee of Hokkaido University Hospital granted specific approval for the inclusion of patients who did not actively refuse to participate as an alternative to obtaining written informed consent.

## Conflicts of Interest

Professor Naoya Sakamoto received lecture fees from Chugai Pharmaceutical Co. Ltd., and research grants from Gilead Sciences Inc. and AbbVie Inc. Dr. Goki Suda received research grants from Gilead Sciences. The authors declare no conflict of interest.

## Supporting information


**Table S1:** Baseline patient characteristics of patients with HCC stratified by the presence or absence of cachexia and sarcopenia.
**Table S2:** Baseline patient characteristics and comparison between patients with and without cachexia/sarcopenia among patients evaluated for overall survival, event onset, transition from compensated cirrhosis to decompensated cirrhosis, and readmissions.
**Table S3:** Baseline characteristics stratified by cachexia/sarcopenia in non‐HCC patients evaluated for survival, disease progression and readmissions.
**Table S4:** Comparative analysis of the occurrence of liver‐related events in patients with and without cachexia/sarcopenia.
**Table S5:** Comparison of the time to readmission for liver‐related events between patients with and without cachexia and sarcopenia using Cox proportional hazards analysis.
**Table S6:** Factors associated with overall survival in non‐HCC patients with liver disease: Cox proportional hazards analysis.
**Table S7:** Comparison of time to liver‐related event between non‐HCC patients with and without cachexia and sarcopenia using Cox proportional hazards analysis.

**Figure S1:** Venn diagram illustrating the definitions of cachexia and sarcopenia and their overlap. BIA, bioelectrical impedance analysis; BMI, body mass index; CRP, C‐reactive protein; CT, computed tomography.
**Figure S2:** Distribution of cachexia and sarcopenia according to HCC stage and systemic therapy in advanced HCC. (a) Proportions of cachexia/sarcopenia categories according to HCC stage. (b) Comparison between patients with and without systemic therapy (ICI or MKI) among those with advanced HCC. (c) Comparison between untreated patients and those receiving MKI therapy among those with advanced HCC. ICI, immune checkpoint inhibitor; BCLC, Barcelona Clinic Liver Cancer; HCC, hepatocellular carcinoma; MKI, multi‐kinase inhibitor.
**Figure S3:** Kaplan–Meier analyses of survival and liver‐related outcomes in non‐HCC patients by cachexia and sarcopenia status. (a) Kaplan–Meier curves for overall survival in non‐HCC patients, stratified by the presence or absence of cachexia and sarcopenia. (b) Kaplan–Meier curves for time to liver‐related events in non‐HCC patients, stratified by the presence or absence of cachexia and sarcopenia. (c) Kaplan–Meier curves for time to transition from compensated to decompensated liver cirrhosis in non‐HCC patients, stratified by the presence or absence of cachexia and sarcopenia. (d) Kaplan–Meier curves for time to readmission in non‐HCC patients, stratified by the presence or absence of cachexia and sarcopenia. CI, confidence interval; HCC, hepatocellular carcinoma; LC, liver cirrhosis; mOS, median overall survival; NR, not reached.

## Data Availability

All data generated or analysed during this study are included in this article and its supplementary data. Further inquiries can be directed to the corresponding authors.
