# Supplementary material for: Prevalence and Prognostic Impact of the Coexistence of Cachexia and Sarcopenia in Patients With Chronic Liver Diseases
Source: J Cachexia Sarcopenia Muscle. 2026 May 5;17(3):e70305. doi: 10.1002/jcsm.70305 (PMC13144545; doi:10.1002/jcsm.70305)
Supplement: Supplementary file 1 — Table S1: Baseline patient characteristics of patients with HCC stratified by the presence or absence of cachexia and sarcopenia. Table S2: Baseline patient characteristics and comparison between patients with and without cachexia/sarcopenia among patients evaluated for overall survival, event onset, transition from compensated cirrhosis to decompensated cirrhosis, and readmissions. Table S3: Baseline characteristics stratified by cachexia/sarcopenia in non‐HCC patients evaluated for survival, disease progression and readmissions. Table S4: Comparative analysis of the occurrence of liver‐related events in patients with and without cachexia/sarcopenia. Table S5: Comparison of the time to readmission for liver‐related events between patients with and without cachexia and sarcopenia using Cox proportional hazards analysis. Table S6: Factors associated with overall survival in non‐HCC patients with liver disease: Cox proportional hazards analysis. Table S7: Comparison of time to liver‐related event between non‐HCC patients with and without cachexia and sarcopenia using Cox proportional hazards analysis. [file JCSM-17-e70305-s001.docx]

**Supporting Information**

***Prevalence and prognostic impact of the coexistence of cachexia and sarcopenia in patients with chronic liver diseases***

Takatsugu Tanaka^1,†^, Goki Suda^1,†,*^, Masatsugu Ohara^1†^, Daisuke Yokoyama¹, [Shoichi Kitano](https://pubmed.ncbi.nlm.nih.gov/?sort=date&term=Kitano+S&cauthor_id=40536752)^1^, Osamu Maehara^2^, Tomoka Yoda^1^, Qingjie Fu^1^, Zijian Yang^1^, Naohiro Yasuura^1^, Akimitsu Meno^1^, Takashi Sasaki^1^, Risako Kohya^1^, Takashi Kitagataya^1^, Naoki Kawagishi^1^, Masato Nakai^1^, Takuya Sho^1^, Shunsuke Ohnishi^2^, and Naoya Sakamoto^1^

^1^ Department of Gastroenterology and Hepatology, Graduate School of Medicine, Hokkaido University, Sapporo, Japan

^2^ Laboratory of Molecular and Cellular Medicine, Faculty of Pharmaceutical Sciences, Hokkaido University, Sapporo, Japan

**^¶^** These authors contributed equally to this work.

Corresponding Author:

Goki Suda

E-mail address: [gsudgast@pop.med.hokudai.ac.jp](mailto:gsudgast@pop.med.hokudai.ac.jp)

**Table S1. Baseline patient characteristics of patients with HCC stratified by the presence or absence of cachexia and sarcopenia.**

| Sample characteristics | Overall  (n = 188) | No cachexia/sarcopenia  (n = 124) | cachexia only  (n = 42) | sarcopenia only  (n = 8) | cachexia  +sarcopenia  (n = 14) | p-value |
| --- | --- | --- | --- | --- | --- | --- |
| Age, Years | 71.0  (42.0-90.0) | 70.0  (42.0-90.0) | 75.0  (46.0-83.0) | 70.5  (58.0-79.0) | 74.0  (48.0-84.0) | 0.171 |
| Sex, Male/Female | 138 / 50 | 92 / 32 | 30 / 12 | 7 / 1 | 9 / 5 | 0.674 |
| BMI, kg/m^2^ | 24.66 (14.86-36.71) | 25.43  (15.16-36.71) | 23.90  (14.97-36.09) | 22.81  (21.63-25.49) | 19.19  (14.86-36.14) | 0.009 |
| Decreased  grip strength, No/Yes | 109 / 79 | 93 / 31 | 16 / 26 | 0 / 8 | 0 / 14 | < 0.001 |
| Appetite loss, No/Yes | 161 / 27 | 114 / 10 | 30 / 12 | 8 / 0 | 9 / 5 | 0.001 |
| PMI, cm^2^/m^2^ | 4.05  (0.71-9.23) | 4.31  (1.41-9.23) | 4.11  (0.71-7.20) | 2.72  (1.62-3.45) | 2.47  (1.21-3.71) | <0.001 |
| Muscle atrophy, No/Yes | 137 / 51 | 100 / 24 | 37 / 5 | 0 / 8 | 0 / 14 | <0.001 |
| History of  diabetes mellitus, No/Yes | 114 / 74 | 81 / 43 | 21 / 21 | 5 / 3 | 7 / 7 | 0.283 |
| Intake of BCAA  supplements, No/Yes | 134 / 54 | 88 / 36 | 30 / 12 | 7 / 1 | 9 / 5 | 0.713 |
| Aetiology |  |  |  |  |  | 0.092 |
| Virus | 84 (44.7%) | 61 | 17 | 2 | 4 |  |
| HBV | 47 (27.0%) | 37 | 7 | 2 | 1 |  |
| HCV | 37 (17.7%) | 24 | 10 | 0 | 3 |  |
| Alcohol | 41 (21.8%) | 29 | 8 | 1 | 3 |  |
| MASH | 47 (25.0%) | 28 | 12 | 2 | 5 |  |
| Others | 16 (8.5%) | 6 | 5 | 3 | 2 |  |
| HBV antiviral therapy  (among HBV-infected  patients) |  |  |  |  |  | 1.000 |
| On nucleos(t)ide  analogue | 43 (22.9%) | 33 | 7 | 2 | 1 |  |
| Without nucleos(t)ide  analogue therapy | 4 (2.1%) | 4 | 0 | 0 | 0 |  |
| History of HCV treatment |  |  |  |  |  | NA |
| SVR after DAA/IFN  therapy | 25 (10.1%) | 16 | 7 | 0 | 2 |  |
| Without SVR | 12 (4.8%) | 8 | 3 | 0 | 1 |  |
| Liver cirrhosis, No/Yes | 45 / 143 | 25 / 99 | 12 / 30 | 4 / 4 | 4 / 10 | 0.202 |
| Child–Pugh class |  |  |  |  |  | 0.905 |
| A | 139 (73.9%) | 92 | 29 | 7 | 11 |  |
| B | 47 (25.0%) | 31 | 12 | 1 | 3 |  |
| C | 2 (1.1%) | 1 | 1 | 0 | 0 |  |
| BCLC stage |  |  |  |  |  | 0.121 |
| 0 | 70 (37.2%) | 51 | 12 | 4 | 3 |  |
| A | 53 (28.2%) | 37 | 12 | 1 | 3 |  |
| B | 33 (17.6%) | 22 | 5 | 2 | 4 |  |
| C | 32 (17.0%) | 14 | 13 | 1 | 4 |  |
| History of systemic  therapy for HCC before enrollment, No/Yes | 159 / 29 | 106 / 18 | 35 / 7 | 6 / 2 | 12 / 2 | 0.872 |
| MKI | 18 (9.6%) | 12 | 4 | 2 | 0 |  |
| ICI-based therapy | 3 (1.6%) | 1 | 1 | 0 | 1 |  |
| MKI+ICI-based therapy | 8 (4.3%) | 5 | 2 | 0 | 1 |  |
| Biochemical analysis |  |  |  |  |  |  |
| Platelet, ×10^4^/μL | 13.30  (2.80-46.40) | 12.15  (2.80-46.40) | 13.35  (5.30-40.00) | 20.40  (5.20-26.80) | 19.10  (6.30-31.00) | 0.092 |
| T-Bil (mg/dL) | 0.80  (0.40-6.70) | 0.90  (0.40-6.70) | 0.80  (0.40-3.40) | 1.00  (0.50-2.70) | 0.75  (0.50-1.80) | 0.456 |
| AST, IU/L | 34.0  (10.0-152.0) | 34.0  (10.0-152.0) | 35.5  (20.0-126.0) | 38.5  (20.0-81.0) | 34.5  (14.0-108.0) | 0.905 |
| ALT, IU/L | 24.0  (5.0-116.0) | 25.0  (5.0-107.0) | 25.0  (10.0-116.0) | 21.5  (12.0-54.0) | 22.0  (7.0-61.0) | 0.684 |
| Serum albumin, g/dL | 3.90  (2.20-4.90) | 3.90  (2.30-4.90) | 3.80  (2.20-4.70) | 3.85  (3.40-4.40) | 3.90  (2.80-4.90) | 0.889 |
| CRP, mg/dL | 0.12  (0.02-6.48) | 0.09  (0.02-2.43) | 0.66  (0.02-6.48) | 0.64  (0.02-2.77) | 0.08  (0.03-4.62) | 0.001 |
| Fib-4 index | 3.84  (0.51-18.67) | 3.86  (0.51-18.67) | 4.12  (1.55-17.53) | 3.02  (1.73-11.11) | 3.37  (1.18-11.37) | 0.695 |
| mALBI grade |  |  |  |  |  | 0.761 |
| 1 | 86 (45.7%) | 57 | 18 | 3 | 8 |  |
| 2a | 44 (23.4%) | 31 | 9 | 3 | 1 |  |
| 2b | 51 (27.1%) | 32 | 12 | 2 | 5 |  |
| 3 | 7 (3.7%) | 4 | 3 | 0 | 0 |  |

Data are presented as numbers or medians (range).

ALT, alanine aminotransferase; AST, aspartate aminotransferase; BCAA, branched-chain amino acids; BCLC, Barcelona Clinic Liver Cancer; BMI, body mass index; CRP, C-reactive protein; DAA, direct-acting antiviral; HBV, hepatitis B virus; HCV, hepatitis C virus; HCC, hepatocellular carcinoma; ICI, immune checkpoint inhibitor; IFN, interferon; mALBI, modified albumin–bilirubin; MASH, metabolic dysfunction-associated steatohepatitis; MKI, multikinase inhibitor; NA, not available; PMI, psoas muscle mass index; T-Bil, total bilirubin; SVR, sustained virologic response.

**Table S2. Baseline patient characteristics and comparison between patients with and without cachexia/sarcopenia among patients evaluated for overall survival, event onset, transition from compensated cirrhosis to decompensated cirrhosis, and readmissions.**

| Sample characteristics | Overall  (n = 246) | No cachexia/sarcopenia  (n = 167) | cachexia only  (n = 44) | sarcopenia only  (n = 13) | cachexia  +sarcopenia  (n = 22) | p-value |
| --- | --- | --- | --- | --- | --- | --- |
| Age, Years | 69.5  (19.0–90.0) | 68.0  (19.0-90.0) | 73.0  (39.0-83.0) | 70.0  (58.0-86.0) | 72.5  (36.0-84.0) | 0.196 |
| Sex, Male/Female | 166 / 80 | 118 / 49 | 28 / 16 | 11 / 2 | 9 / 13 | 0.020 |
| BMI, kg/m^2^ | 24.44  (14.86-42.56) | 25.24  (15.16-36.71) | 23.59  (14.97-42.56) | 23.00  (21.63-25.76) | 19.49  (14.86-36.14) | <0.001 |
| Decreased  grip strength, No/Yes | 150 / 96 | 131 / 36 | 19 / 25 | 0 / 13 | 0 / 22 | <0.001 |
| Appetite loss, No/Yes | 208 / 38 | 154 / 13 | 28 / 16 | 12 / 1 | 14 / 8 | <0.001 |
| PMI, cm^2^/m^2^ | 3.86  (0.71-9.23) | 4.13  (1.41-9.23) | 3.91  (0.71-7.20) | 2.93  (0.91-3.73) | 2.05  (1.05-3.71) | <0.001 |
| Muscle atrophy, No/Yes | 168 / 78 | 131 / 36 | 37 / 7 | 0 /13 | 0 / 22 | <0.001 |
| History of  diabetes mellitus,  No/Yes | 160 / 86 | 117 / 50 | 24 / 20 | 5 / 8 | 14 / 8 | 0.045 |
| Intake of BCAA  supplements, No/Yes | 163 / 83 | 112 / 55 | 30 / 14 | 8 / 5 | 13 / 9 | 0.860 |
| Aetiology |  |  |  |  |  | 0.244 |
| Virus | 111 (45.1%) | 82 | 19 | 5 | 5 |  |
| HBV | 67 (27.0%) | 55 | 8 | 2 | 2 |  |
| HCV | 44 (17.7%) | 27 | 11 | 3 | 3 |  |
| Alcohol | 52 (21.1%) | 36 | 10 | 1 | 5 |  |
| MASH | 55 (22.4%) | 35 | 9 | 4 | 7 |  |
| Others | 28 (11.4%) | 14 | 6 | 3 | 5 |  |
| HBV antiviral therapy  (among HBV-infected  patients) |  |  |  |  |  | 0.733 |
| On nucleos(t)ide  analogue | 60 (24.2%) | 48 | 8 | 2 | 2 |  |
| Without nucleos(t)ide  analogue therapy | 7 (2.8%) | 7 | 0 | 0 | 0 |  |
| History of HCV  treatment |  |  |  |  |  | 0.506 |
| SVR after DAA/IFN  therapy | 27 (10.9%) | 18 | 7 | 1 | 1 |  |
| Without SVR | 17 (6.9%) | 9 | 4 | 2 | 2 |  |
| Liver cirrhosis, No/Yes | 38 / 208 | 22 / 145 | 10 / 34 | 3 / 10 | 3 / 19 | 0.379 |
| Child–Pugh class |  |  |  |  |  | 0.270 |
| A | 162 (65.9%) | 116 | 26 | 8 | 12 |  |
| B | 73 (29.7%) | 46 | 15 | 3 | 9 |  |
| C | 11 (4.5%) | 5 | 3 | 2 | 1 |  |
| HCC, No/Yes | 88 / 158 | 63 / 104 | 9 / 35 | 7 / 6 | 9 / 13 | 0.076 |
| BCLC stage |  |  |  |  |  | 0.093 |
| 0 | 58 (36.7%) | 43 | 10 | 3 | 2 |  |
| A | 47 (29.7%) | 31 | 12 | 1 | 3 |  |
| B | 28 (17.7%) | 20 | 3 | 1 | 4 |  |
| C | 25 (15.8%) | 10 | 10 | 1 | 4 |  |
| History of systemic  therapy for HCC before enrollment, No/Yes | 134 / 24 | 90 / 14 | 29 / 6 | 4 / 2 | 11 / 2 | 0.598 |
| MKI | 18 (7.3%) | 12 | 4 | 2 | 0 |  |
| ICI-based therapy | 2 (0.8%) | 0 | 1 | 0 | 1 |  |
| MKI+ICI-based therapy | 4 (1.6%) | 2 | 1 | 0 | 1 |  |
| History of systemic  therapy for HCC, No/Yes | 92 / 66 | 63 / 41 | 20 / 15 | 3 / 3 | 6 / 7 | 0.753 |
| MKI | 38 (15.3%) | 26 | 8 | 2 | 2 |  |
| ICI-based therapy | 8 (3.2%) | 6 | 1 | 0 | 1 |  |
| MKI+ICI-based therapy | 20 (8.1%) | 9 | 6 | 1 | 4 |  |
| Biochemical analysis |  |  |  |  |  |  |
| Platelet, ×10^4^/μL | 11.95  (1.90-66.50) | 11.30  (2.80-46.40) | 13.55 (1.90-40.00) | 9.60  (5.20-39.70) | 14.95  (6.30-66.50) | 0.060 |
| T-Bil (mg/dL) | 0.90  (0.30-18.40) | 0.90  (0.40-6.70) | 0.80  (0.40-18.40) | 0.90  (0.30-2.70) | 0.80  (0.30-2.90) | 0.437 |
| AST, IU/L | 33.50  (10.00-601.00) | 32.0  (10.0-601.0) | 35.0  (21.0-155.0) | 41.0  (24.0-100.0) | 38.0  (15.0-108.0) | 0.067 |
| ALT, IU/L | 24.50  (5.00-613.00) | 25.0  (5.0-613.0) | 25.0  (7.0-214.0) | 23.0  (11.0-61.0) | 23.5  (10.0-72.0) | 0.843 |
| Serum albumin, g/dL | 3.90  (2.20-5.00) | 3.90  (2.20-5.00) | 3.85  (2.20-4.70) | 3.60  (2.50-4.40) | 3.65  (2.20-4.90) | 0.129 |
| CRP, mg/dL | 0.09  (0.02-6.30) | 0.08  (0.02-3.17) | 0.70  (0.02-6.30) | 0.13  (0.02-2.77) | 0.35  (0.02-4.62) | <0.001 |
| Fib-4 index | 3.96  (0.38-31.20) | 3.91  (0.51-18.67) | 4.12  (1.24-31.20) | 5.84  (1.24-13.74) | 3.83  (0.38-11.37) | 0.397 |
| mALBI grade |  |  |  |  |  | 0.340 |
| 1 | 110 (44.4%) | 77 | 20 | 5 | 8 |  |
| 2a | 51 (20.6%) | 38 | 8 | 2 | 3 |  |
| 2b | 71 (28.6%) | 46 | 11 | 4 | 10 |  |
| 3 | 14 (5.6%) | 6 | 5 | 2 | 1 |  |

Data are presented as numbers or medians (range).

ALT, alanine aminotransferase; AST, aspartate aminotransferase; BCAA, branched-chain amino acids; BCLC, Barcelona Clinic Liver Cancer; BMI, body mass index; CRP, C-reactive protein; DAA, direct-acting antiviral; HBV, hepatitis B virus; HCV, hepatitis C virus; HCC, hepatocellular carcinoma; ICI, immune checkpoint inhibitor; IFN, interferon; mALBI, modified albumin–bilirubin; MASH, metabolic dysfunction-associated steatohepatitis; MKI, multikinase inhibitor; PMI, psoas muscle mass index; T-Bil, total bilirubin; SVR, sustained virologic response.

**Table S3. Baseline characteristics stratified by cachexia/sarcopenia in non-HCC patients evaluated for survival, disease progression, and readmissions.**

| Sample characteristics | Overall  (n = 88) | No cachexia/sarcopenia  (n = 63) | cachexia only  (n = 9) | sarcopenia only  (n = 7) | cachexia  +sarcopenia  (n = 9) | p-value |
| --- | --- | --- | --- | --- | --- | --- |
| Age, Years | 66.0  (19.0–86.0) | 67.0  (19.0-84.0) | 59.0  (39.0-66.0) | 70.0  (59.0-86.0) | 72.0  (36.0-76.0) | 0.028 |
| Sex, Male/Female | 49 / 39 | 39 / 24 | 3 / 6 | 6 / 1 | 1 / 8 | 0.006 |
| BMI, kg/m^2^ | 24.32  (16.55-42.56) | 24.89  (18.40-33.88) | 22.34  (19.41-42.56) | 24.62  (22.09-25.76) | 19.12  (16.55-27.83) | 0.001 |
| Decreased  grip strength, No/Yes | 53 / 35 | 48 / 15 | 5 / 4 | 0 / 7 | 0 / 9 | < 0.001 |
| Appetite loss, No/Yes | 76 / 12 | 60 / 3 | 4 / 5 | 6 / 1 | 6 / 3 | < 0.001 |
| PMI, cm^2^/m^2^ | 3.50  (0.91-6.83) | 3.83  (1.97-6.83) | 3.11  (1.52-4.99) | 2.97  (0.91-3.73) | 1.48  (1.05-3.23) | < 0.001 |
| Muscle atrophy, No/Yes | 56 / 32 | 50 / 13 | 6 / 3 | 0 / 7 | 0 / 9 | < 0.001 |
| History of  diabetes mellitus, No/Yes | 57 / 31 | 45 / 18 | 4 / 5 | 1 / 6 | 7 / 2 | 0.010 |
| Intake of BCAA  supplements, No/Yes | 53 / 35 | 40 / 23 | 5 / 4 | 3 / 4 | 5 / 4 | 0.723 |
| Aetiology |  |  |  |  |  | 0.841 |
| Virus | 36 (40.9%) | 29 | 2 | 3 | 2 |  |
| HBV | 23 (26.1%) | 21 | 1 | 0 | 1 |  |
| HCV | 13 (14.8%) | 8 | 1 | 3 | 2 |  |
| Alcohol | 18 (20.5%) | 12 | 3 | 1 | 2 |  |
| MASH | 19 (21.6%) | 13 | 2 | 2 | 2 |  |
| Others | 15 (17.0%) | 9 | 2 | 1 | 3 |  |
| HBV antiviral therapy  (among HBV-infected  patients) |  |  |  |  |  | 1.000 |
| On nucleos(t)ide  analogue | 20 (22.7%) | 18 | 1 | 0 | 1 |  |
| Without nucleos(t)ide  analogue therapy | 3（3.4%） | 3 | 0 | 0 | 0 |  |
| History of HCV treatment |  |  |  |  |  | 0.233 |
| SVR after DAA/IFN  therapy | 7 (8.0%) | 6 | 0 | 1 | 0 |  |
| Without SVR | 6 (6.8%) | 2 | 1 | 2 | 1 |  |
| Liver cirrhosis,  No/Yes | 0 / 88 | 0 / 63 | 0 / 9 | 0 / 7 | 0 / 9 | NA |
| Child–Pugh class |  |  |  |  |  | 0.011 |
| A | 47 (53.4%) | 41 | 1 | 3 | 2 |  |
| B | 32 (36.4%) | 18 | 6 | 2 | 6 |  |
| C | 9 (10.2%) | 4 | 2 | 2 | 1 |  |
| Biochemical analysis |  |  |  |  |  |  |
| Platelet, ×10^4^/μL | 11.10  (1.90-66.50) | 11.10  (3.10-37.80) | 14.20  (1.90-22.50) | 8.10  (6.00-39.70) | 13.10  (8.40-66.50) | 0.349 |
| T-Bil (mg/dL) | 1.00  (0.30-18.40) | 1.10  (0.40-6.10) | 1.80  (0.70-18.40) | 0.80  (0.30-1.60) | 0.80  (0.30-2.90) | 0.102 |
| AST, IU/L | 32.0  (15.0-601.0) | 29.0  (16.0-601.0) | 35.0  (21.0-155.0) | 39.0  (26.0-100.0) | 47.0  (15.0-98.0) | 0.076 |
| ALT, IU/L | 24.5  (7.0-613.0) | 21.0  (9.0-613.0) | 26.0  (7.0-214.0) | 23.0  (11.0-61.0) | 27.0  (11.0-72.0) | 0.493 |
| Serum albumin, g/dL | 3.80  (2.20-5.00) | 3.90  (2.20-5.00) | 3.50  (2.60-4.30) | 3.30  (2.50-4.10) | 3.00  (2.20-4.20) | 0.006 |
| CRP, mg/dL | 0.08  (0.02-3.17) | 0.06  (0.02-3.17) | 0.43  (0.02-1.86) | 0.06  (0.02-2.37) | 0.47  (0.02-1.69) | 0.010 |
| Fib-4 index | 4.30  (0.38-31.20) | 4.21  (0.63-15.24) | 3.65  (1.24-31.20) | 7.29  (1.24-13.74) | 4.52  (0.38-8.42) | 0.366 |
| mALBI grade |  |  |  |  |  | 0.085 |
| 1 | 34 (38.6%) | 28 | 2 | 3 | 1 |  |
| 2a | 16 (18.2%) | 13 | 1 | 0 | 2 |  |
| 2b | 29 (33.0%) | 19 | 3 | 2 | 5 |  |
| 3 | 9 (10.2%) | 3 | 3 | 2 | 1 |  |

Data are presented as numbers or medians (range).

ALT, alanine aminotransferase; AST, aspartate aminotransferase; BCAA, branched-chain amino acids; BMI, body mass index; CRP, C-reactive protein; DAA, direct-acting antiviral; HBV, hepatitis B virus; HCV, hepatitis C virus; HCC, hepatocellular carcinoma; IFN, **interferon**; mALBI, modified albumin–bilirubin; MASH, metabolic dysfunction-associated steatohepatitis; NA, not available; PMI, psoas muscle mass index; T-Bil, total bilirubin; SVR, sustained virologic response.

**Table S4.** **Comparative analysis of the occurrence of liver-related events in patients with and without cachexia/sarcopenia.**

| Sample characteristics | Overall  (n = 246) | No  cachexia/sarcopenia  (n = 167) | cachexia only  (n = 44) | sarcopenia only  (n = 13) | cachexia  +sarcopenia  (n = 22) | P-value |
| --- | --- | --- | --- | --- | --- | --- |
| Liver-related event | 64 | 40 | 11 | 3 | 10 | 0.189 |
| Hepatic encephalopathy | 15 | 8 | 5 | 0 | 2 |  |
| Oesophageal /Gastric  varices rupture | 6 | 4 | 1 | 1 | 0 |  |
| Worsening ascites | 49 | 29 | 10 | 2 | 8 |  |
| SBP | 6 | 2 | 1 | 0 | 3 |  |
| Portal vein thrombosis | 12 | 10 | 1 | 1 | 0 |  |
| LC (Compensated/Decompensated  /Compensated→Decompensated) | 144 / 70 / 32 | 100 / 44 / 23 | 28 / 12 / 4 | 6 / 7 / 0 | 10 / 7 / 5 | 0.208 |
| Readmission（No/Yes） | 163 / 83 | 115 / 52 | 27 / 17 | 9 / 4 | 12 / 10 | 0.497 |

Data are presented as numbers or medians (range).

HCC, hepatocellular carcinoma; LC, liver cirrhosis; SBP, spontaneous bacterial peritonitis.

**Table S5.** **Comparison of the time to readmission for liver-related events between patients with and without cachexia and sarcopenia using Cox proportional hazards analysis.**

|  | Univariable analysis | | Multivariable analysis | | | | | |
| --- | --- | --- | --- | --- | --- | --- | --- | --- |
|  | HR (95% CI) | P-value | HR (95% CI)  Model A | P-value | HR (95% CI)  Model B | P-value | HR (95% CI)  Model C | P-value |
| Age, Years | 0.975  (0.958-0.992) | 0.004 | 0.982  (0.962-1.002) | 0.079 | 0.977  (0.957-0.997) | 0.027 | 0.979  (0.960-0.999) | 0.038 |
| Sex, Women | 1.505  (0.968-2.341) | 0.070 | 1.211  (0.736-1.993) | 0.451 | 1.166  (0.705-1.927) | 0.550 | 1.251  (0.765-2.046) | 0.372 |
| No cachexia/sarcopenia | 1 (Reference) |  | 1 (Reference) |  | 1 (Reference) |  | 1 (Reference) |  |
| cachexia only | 1.639  (0.943-2.848) | 0.080 | 1.276  (0.714-2.279) | 0.411 | 1.237  (0.690-2.218) | 0.474 | 1.295  (0.730-2.298) | 0.376 |
| sarcopenia only | 1.271  (0.459-3.523) | 0.644 |  |  |  |  |  |  |
| cachexia+sarcopenia | 2.344  (1.186-4.633) | 0.014 | 1.840  (0.889-3.811) | 0.101 | 2.077  (1.006-4.288) | 0.048 | 2.208  (1.072-4.547) | 0.032 |
| Aetiology, Virus | 1 (Reference) |  | 1 (Reference) |  | 1 (Reference) |  |  |  |
| Alcohol | 1.108  (0.611-2.008) | 0.736 |  |  |  |  |  |  |
| MASH | 1.902  (1.108-3.262) | 0.020 | 1.159  (0.632-2.125) | 0.635 | 1.147  (0.629-2.089) | 0.655 | 1.291  (0.711-2.342) | 0.401 |
| Others | 1.989  (0.980-4.036) | 0.057 | 1.099  (0.489-2.468) | 0.820 | 0.780  (0.336-1.813) | 0.564 | 0.896  (0.397-2.021) | 0.791 |
| History of diabetes mellitus | 1.958  (1.265-3.033) | 0.003 | 1.774  (1.096-2.872) | 0.020 | 1.685  (1.035-2.743) | 0.036 | 1.756  (1.083-2.846) | 0.022 |
| Child–Pugh class A  versus B, C | 2.454  (1.582-3.805) | < 0.001 | 1.863  (1.160-2.993) | 0.010 |  |  |  |  |
| HCC | 0.769  (0.497-1.190) | 0.238 |  |  |  |  |  |  |
| Serum albumin, g/dL | 0.373 (0.265-0.526) | < 0.001 |  |  | 0.424  (0.297-0.607) | < 0.001 |  |  |
| mALBI  grade 1–2a versus 2b–3 | 2.690 (1.732-4.177) | < 0.001 |  |  |  |  | 2.387  (1.506-3.785) | < 0.001 |

CI, confidence interval; HR, hazard ratio; HCC, hepatocellular carcinoma; mALBI, modified albumin–bilirubin; MASH, metabolic dysfunction-associated steatohepatitis.

**Table S6. Factors associated with overall survival in non-HCC patients with liver disease: Cox proportional hazards analysis.**

|  | Univariable analysis | | Multivariable analysis | |
| --- | --- | --- | --- | --- |
|  | HR (95% CI) | P-value | HR (95% CI) | P-value |
| Age, Years | 1.004 (0.963-1.047) | 0.855 |  |  |
| Sex, Women | 1.108 (0.436-2.815) | 0.830 |  |  |
| No cachexia/sarcopenia | 1 (Reference) |  | 1 (Reference) |  |
| cachexia only | 2.296 (0.617-8.540) | 0.215 |  |  |
| sarcopenia only | 5.903 (1.542-22.59) | 0.010 | 7.683 (1.866-31.640) | 0.005 |
| cachexia+sarcopenia | 3.895 (1.002-15.150) | 0.050 | 4.931 (1.212-20.070) | 0.026 |
| Aetiology, Virus | 2.022 (0.798-5.122) | 0.138 |  |  |
| Alcohol | 1 (Reference) |  | 1 (Reference) |  |
| MASH | 2.524 (0.913-6.975) | 0.074 | 2.146 (0.763-6.037) | 0.148 |
| Others | 0.671 (0.138-3.251) | 0.620 |  |  |
| History of diabetes mellitus | 0.507 (0.062-4.154) | 0.527 |  |  |
| Child–Pugh class A versus B, C | 2.014 (0.785-5.167) | 0.145 |  |  |

CI, confidence interval; HR, hazard ratio; HCC, hepatocellular carcinoma; MASH, metabolic dysfunction-associated steatohepatitis

**Table S7. Comparison of time to liver-related event between non-HCC patients with and without cachexia and sarcopenia using Cox proportional hazards analysis.**

|  | Univariable analysis | | Multivariable analysis | | |
| --- | --- | --- | --- | --- | --- |
|  | HR (95% CI) | P-value | | HR (95% CI) | P-value |
| Age, Years | 0.993 (0.967-1.020) | 0.616 | |  |  |
| Sex, Women | 1.822 (0.905-3.668) | 0.093 | | 1.303 (0.563-3.016) | 0.537 |
| No cachexia/sarcopenia | 1 (Reference) |  | | 1 (Reference) |  |
| cachexia only | 3.616 (1.398-9.353) | 0.008 | | 1.939 (0.657-5.722) | 0.231 |
| sarcopenia only | 2.616 (0.753-9.088) | 0.130 | |  |  |
| cachexia+sarcopenia | 5.710 (2.307-14.130) | < 0.001 | | 3.305 (1.104-9.894) | 0.032 |
| Aetiology, Virus | 1.926 (0.960-3.863) | 0.065 | | 1.433 (0.677-3.035) | 0.348 |
| Alcohol | 1 (Reference) |  | |  |  |
| MASH | 1.446 (0.559-3.737) | 0.447 | |  |  |
| Others | 1.558 (0.620-3.916) | 0.346 | |  |  |
| History of diabetes mellitus | 1.901 (0.696-5.196) | 0.210 | |  |  |
| Child–Pugh class A versus B, C | 3.486 (1.634-7.439) | 0.001 | | 2.262 (0.958-5.340) | 0.063 |

CI, confidence interval; HR, hazard ratio; HCC, hepatocellular carcinoma; MASH, metabolic dysfunction-associated steatohepatitis
