# Supplementary material for: Prevalence and Prognostic Impact of the Coexistence of Cachexia and Sarcopenia in Patients With Chronic Liver Diseases
Source: J Cachexia Sarcopenia Muscle. 2026 May 5;17(3):e70305. doi: 10.1002/jcsm.70305 (PMC13144545; doi:10.1002/jcsm.70305)
Supplement: Supplementary file 2 — Figure S1: Venn diagram illustrating the definitions of cachexia and sarcopenia and their overlap. BIA, bioelectrical impedance analysis; BMI, body mass index; CRP, C‐reactive protein; CT, computed tomography. Figure S2: Distribution of cachexia and sarcopenia according to HCC stage and systemic therapy in advanced HCC. (a) Proportions of cachexia/sarcopenia categories according to HCC stage. (b) Comparison between patients with and without systemic therapy (ICI or MKI) among those with advanced HCC. (c) Comparison between untreated patients and those receiving MKI therapy among those with advanced HCC. ICI, immune checkpoint inhibitor; BCLC, Barcelona Clinic Liver Cancer; HCC, hepatocellular carcinoma; MKI, multi‐kinase inhibitor. Figure S3: Kaplan–Meier analyses of survival and liver‐related outcomes in non‐HCC patients by cachexia and sarcopenia status. (a) Kaplan–Meier curves for overall survival in non‐HCC patients, stratified by the presence or absence of cachexia and sarcopenia. (b) Kaplan–Meier curves for time to liver‐related events in non‐HCC patients, stratified by the presence or absence of cachexia and sarcopenia. (c) Kaplan–Meier curves for time to transition from compensated to decompensated liver cirrhosis in non‐HCC patients, stratified by the presence or absence of cachexia and sarcopenia. (d) Kaplan–Meier curves for time to readmission in non‐HCC patients, stratified by the presence or absence of cachexia and sarcopenia. CI, confidence interval; HCC, hepatocellular carcinoma; LC, liver cirrhosis; mOS, median overall survival; NR, not reached. [file JCSM-17-e70305-s002.pptx]

## Slide 1
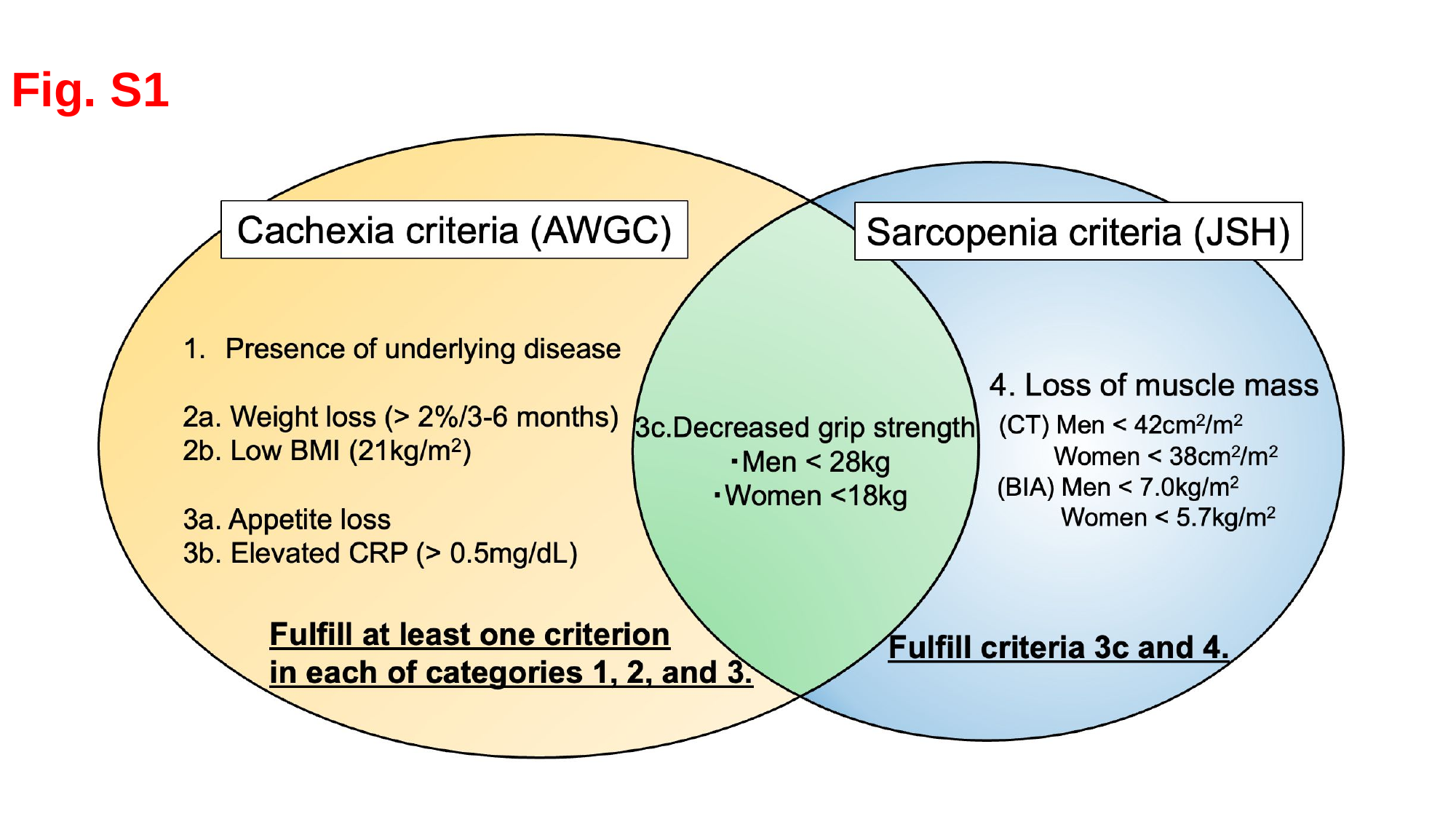

# Fig. S1

## Slide 2
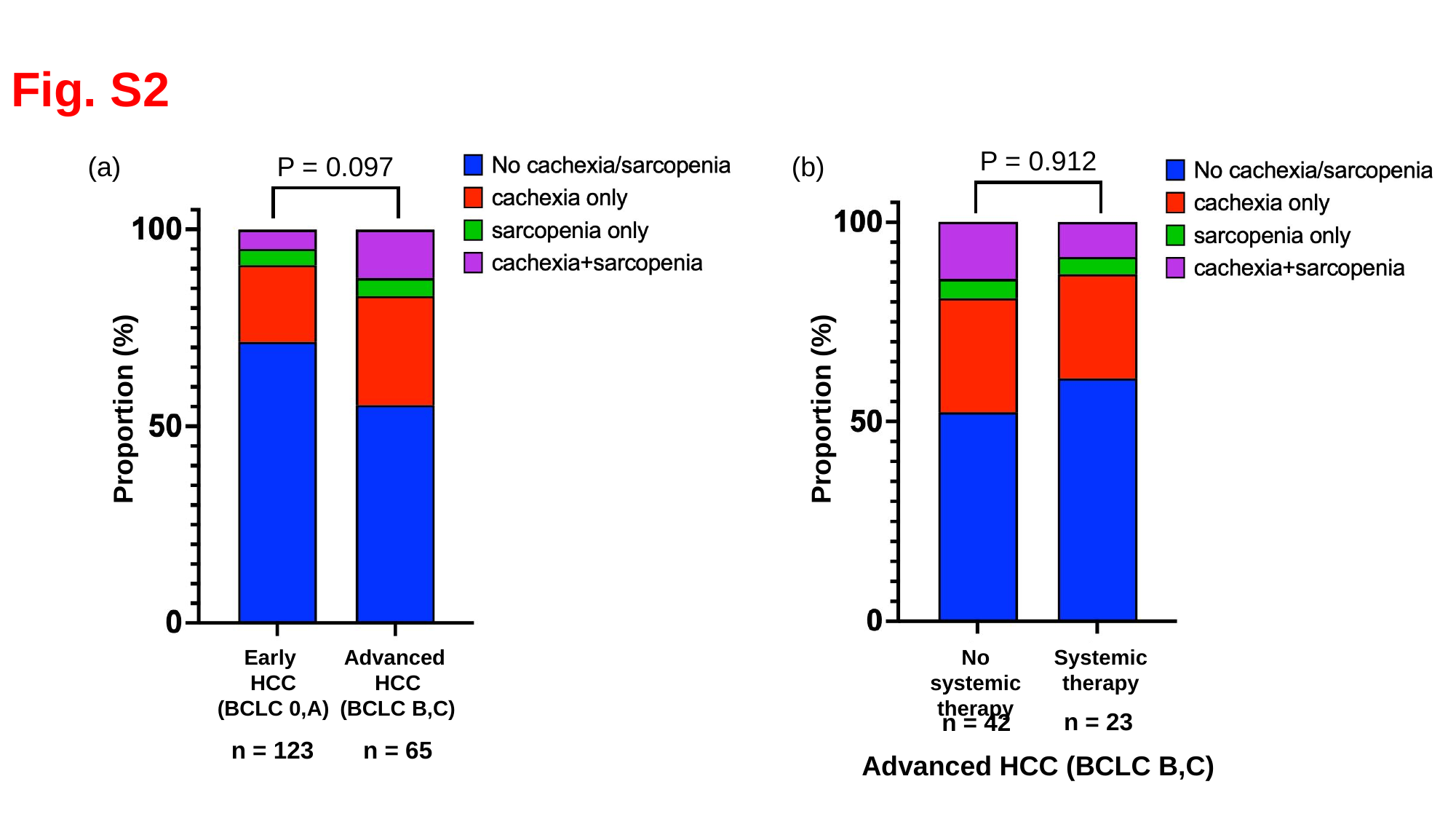

# Fig. S2
P = 0.912
P = 0.097
(b)
(a)
Proportion (%)
Proportion (%)
Early
HCC
(BCLC 0,A)
Advanced
HCC
(BCLC B,C)
No systemic therapy
Systemic therapy
n = 23
n = 42
n = 123
n = 65
Advanced HCC (BCLC B,C)

## Slide 3
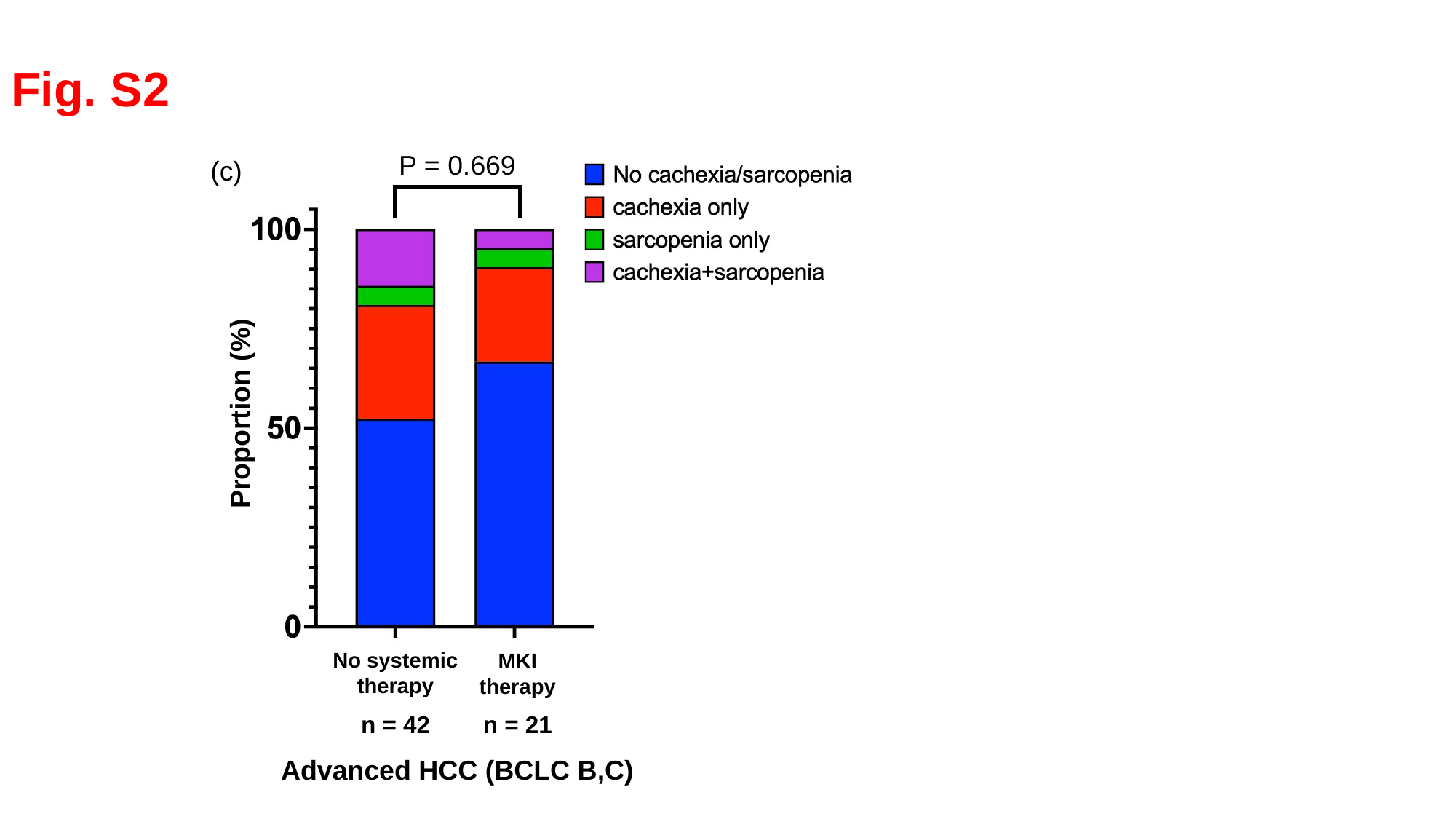

# Fig. S2
P = 0.669
(c)
Proportion (%)
No systemic therapy
MKI therapy
n = 21
n = 42
Advanced HCC (BCLC B,C)

## Slide 4
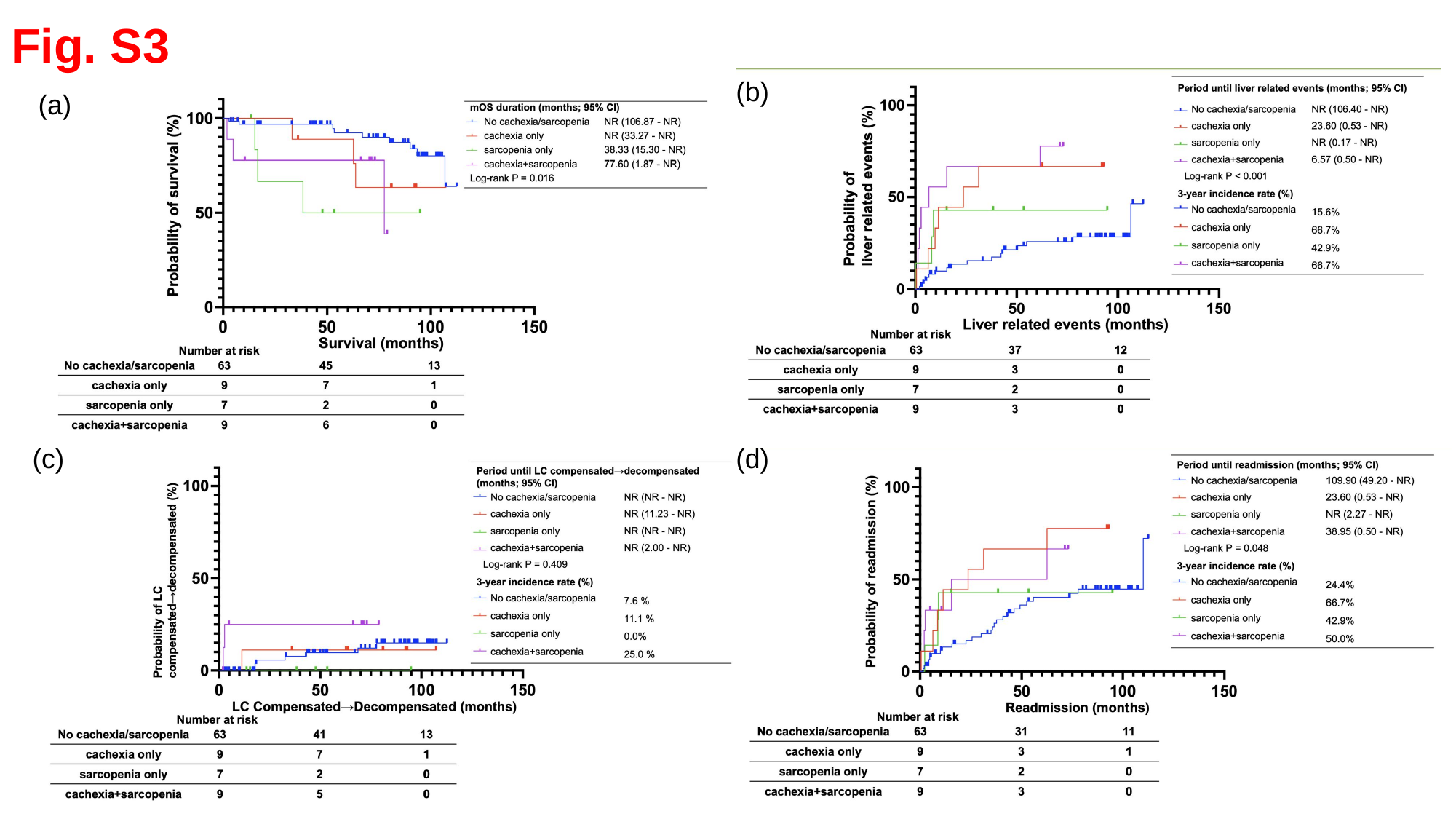

# Fig. S3
(b)
(a)
(c)
(d)
